# Understanding the adoption of new drugs decided by several stakeholders in the South Korean market: a nonparametric event history analysis

**DOI:** 10.1186/s13561-018-0216-4

**Published:** 2018-12-04

**Authors:** Kyung-Bok Son

**Affiliations:** College of Pharmacy, Ewha Womans University, 52Ewhayeodae-gil, Seodaemun-gu, Seoul, 03760 South Korea

**Keywords:** New drug, Reimbursement decision, Delay in access, Event history model

## Abstract

**Background:**

Regulatory approval and reimbursement decisions are necessary if new drugs are to become accessible in a timely manner. However, the process of regulatory approval and the establishment of reimbursement decisions varies across countries. This study aims to analyze the duration between regulatory approval and reimbursement decision for new drugs and to evaluate various factors affecting the timely availability of new medicines in the Korean market. The duration was subdivided into regulatory approval–reimbursement application and reimbursement application–reimbursement decision. We used pharmaceutical approval data to identify new medicines, retrieved documents from the pharmaceutical benefits committee to collect information on reimbursement decision, and applied a non-parametric event history model.

**Results:**

A total of 128 new medicines applied for reimbursement decision, including 85 drugs between 2007 and 2013 and 43 drugs between 2014 and 2016. Delays in access to new medicines occurred at various levels, and various factors affected in different durations. In proportional hazard model, the second period shortened all durations in the models. Biologics and clinically improved drugs were the factor that delayed the duration of regulatory approval–reimbursement application, while uncertain drugs in clinical effectiveness and ATC J or L delayed the duration of reimbursement application–reimbursement decision.

**Conclusions:**

The duration between regulatory approval and reimbursement decision has decreased, and the main cause of the delay has changed. For instance, the proportion of reimbursement trial–reimbursement decision in the total duration was 62.9% (18.39 months out of 29.24 months) in the first period, while the proportion of regulatory approval–reimbursement trial in the total duration was 64.2% (8.6 months out of 13.40 months) in the second period. A series of policies to reinforce access to medicines after 2014 has been effective for the timely availability of new medicines, including both prompt reimbursement application decided by manufacturers and timely review process by the authorities.

## Background

Regulatory approval and reimbursement decisions are necessary if new drugs are to become accessible in a timely manner [1–3]. However, the process of regulatory approval and the establishment of reimbursement decisions varies across countries. In 1995, the European Union adopted the “Centralized Procedure” to evaluate new drugs and since then, has granted regulatory approval that is valid in all EU member states [4]. However, each member state still individually manages its own pricing and reimbursement decision [5–7]. Some countries, notably including England, have implemented a health technology assessment for their pricing and reimbursement decision [8]. Furthermore, many countries have newly adopted an economic evaluation of new medicines to control their increasing health care expenditures [9, 10].

In line with this trend, the South Korean government (hereafter Korea) adopted health technology assessments for reimbursement decisions concerning new medicines in 2006 [10–14]. Specifically, the government implemented a “drug expenditure rationalization plan” (the Plan) to rationalize pharmaceutical expenditures. A positive list system (PLS) was then introduced in 2007. There have been concerns about “delay in access to new medicines” since health technology assessments adopted in Korea [3], because patients must wait until the pricing and reimbursement decision process is concluded before they can utilize medicines under the National Health Insurance Service (NHIS) [15–17]. To address these concerns, the government recently introduced a series of policies to reinforce access to new medicines [10, 18]. Specifically, the government introduced risk sharing agreements [19, 20], which are similar to managed entry schemes in European countries [21–23], and adopted a flexible incremental cost-effectiveness ratio (ICER) threshold in 2013 [10]. In addition, exemption from price negotiations between manufacturers and the NHIS and exemption from the health technology assessment for selected new medicines were newly introduced in 2015 [10].

We are interested in the duration from regulatory approval to reimbursement decision for new drugs in the Korean market. This topic is noteworthy because there are many decision points determined by various stakeholders, including manufacturers, the Ministry of Food and Drug Safety (MFDS), the Health Insurance Review and Assessment Service (HIRA), the NHIS, and the Ministry of Health and Welfare (MOHW), in the new system.

### The regulatory approval and reimbursement process in Korea

The MFDS approves new medicines based on submitted data demonstrating the safety and efficacy of the drug. After regulatory approval, manufacturers can decide whether to apply for reimbursement in the Korean market, but reimbursement by the NHIS is essential in manufacturers want to penetrate and expand the pharmaceutical market. Manufacturers who want their new drugs to be eligible for reimbursement must submit the applications and related dossiers to the HIRA. The staff at HIRA review these dossiers, and assess the clinical-effectiveness and cost-effectiveness of the drug. Specifically, the Benefit Criteria Advisory Committee reviews and sets benefit criteria considering clinical-effectiveness, and the Economic Evaluation Subcommittee reviews whether an economic evaluation is necessary and the submitted data are valid. Then, the Pharmaceutical Benefits Committee (PBC) appraises whether the drug can be reimbursed. It should be noted that the PBC considers various factors before recommending the listing of a drug: clinical-effectiveness, cost-effectiveness, budget impact, and reimbursement and pricing of the drug in other countries [24]. There is no explicit threshold for the cost-effectiveness of new medicines provided by the HIRA [25].

After the PBC recommends listing the drug, the NHIS negotiates price and expected volumes with the manufacturer [26]. Negotiations are essential for new drugs to be reimbursed by the NHIS. The NHIS considers comparator drugs, number of patients with the related diseases, reimbursement criteria, clinical-effectiveness, budget impact, and reimbursement and pricing of the drug in other countries. When the negotiation reaches an agreement, the Health Insurance Policy Council (HIPC) reviews the results. If the results are approved by the HIPC, the MOHW makes a decision and announces the maximum allowable listing price to the public. If the price negotiation fails, the drug cannot be reimbursed. However, if a drug is deemed medically necessary, additional steps are taken to ensure their availability. Medically necessary drugs are designated by regulations and fit the following criteria [27]: a) there are no alternative treatments for the drug; b) the drug is used for severe life-threatening disease; c) the drug is used for a very rare disease and is considered necessary to treat the rare disease; and d) the health benefits of the drug are supported by evidence. The Benefit Coordination Committee, which is under the jurisdiction of the MOHW, determines the price by authority, and the MOHW reimburses the medically necessary drug accordingly [10–12, 17, 26].

Given these various players and processes, delays in access to medicines may occur at various points [3, 28]. Sometimes, a manufacturer may intentionally delay launching new medicines in the market even after regulatory approval, specifically in a low-price market [3], while the pricing and reimbursement authority, including the HIRA and the NHIS may cause a delay if the submitted dossiers are incomplete or do not contain enough information for decision making [3].

In this study, we aim to analyze the duration between regulatory approval and reimbursement decision for new medicines in the Korean market and to evaluate various factors affecting the timely availability of new medicines. In addition, we selected several decision points and subdivided the duration into regulatory approval–reimbursement application and reimbursement application–reimbursement decision to identify the prolonging or abbreviating factors, including the strategic behavior of manufacturers, in the reimbursement decision for new drugs.

## Methods

### Data sources

The MFDS designates a “new drug,” which means a drug of new materials; a chemical structure or the construction of a substance that is wholly new; or a drug of composite medication containing new materials as the effective ingredient “Pharmaceutical Affairs Act. Article 2 (Definitions)”. Building upon this definition, we defined new medicines based on their active ingredient. Specifically, we selected new medicines designated by the MFDS between 2007 and 2016 for this study. Note that the new PLS system was introduced in 2007. The data set used in this study was obtained from publicly available information prepared by the MFDS.

In addition, we retrieved documents from the PBC posted on the HIRA website to collect information on reimbursement decisions. Specifically, we found information on reimbursement recommendations by the PBC and the date when the application was reviewed. It should be noted that manufacturers can decide whether to apply for reimbursement under the PLS. Thus, we excluded new medicines that have not had applications made for reimbursement.

### Models

This study address the duration from regulatory approval to reimbursement decisions for new drugs in the Korean market. However, there are many decision points in this process that are determined by various actors [10]: the MFDS approves new medicines, a manufacturer decides whether to apply for reimbursement, the HIRA reviews the submitted dossiers, the NHIS negotiates the price with the manufacturers, and the MOHW determines final reimbursement including price. Thus, we subdivided the duration into regulatory approval–reimbursement application and reimbursement application–reimbursement decision.

**Fig. 1:**
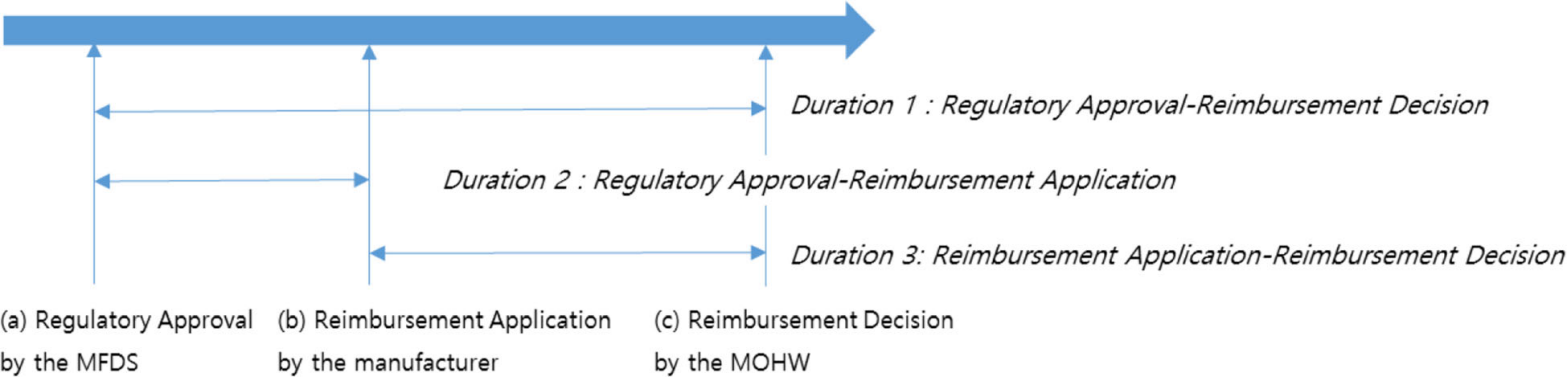
The process between regulatory approval and reimbursement decision with various decision points

Specifically, we used the data set prepared by the MFDS to identify (a) the regulatory approval date; retrieved documents from the PBC posted on the HIRA website to collect information on (b) the first reimbursement trial date; and searched the HIRA website to identify (c) the reimbursement decision date (Fig. 1). Accordingly, we constructed the dependent variable as the time difference between (a), (b), and (c): regulatory approval–reimbursement decision (duration 1), regulatory approval–reimbursement application (duration 2), and reimbursement application–reimbursement decision (duration 3). Data management and analysis were performed using R statistical software (version 3.4.1). Statistical significance was defined as *p*-values under 0.05.

We should note that our observations are right-censored. Therefore, we applied an event history model for a statistical estimation of the duration. The model, also known as duration model, estimates the duration until an event occurs which in our case is the reimbursement decision, and identifies abbreviating or prolonging factors. We applied Kaplan-Meier survival estimates as a univariate tool and the proportional hazards model for a multivariate approach to determine the relative impact of the specific factors on various durations. In our model, we included five discrete factors: manufacturing type; product type, including chemicals and biologics; the Anatomical Therapeutic Chemical Classification (ATC); clinical effectiveness of the medicine as decided by the PBC; and the period, such as before 2014 or after 2014.

In January 2012, the MOHW reported a “pharmaceutical industry competitiveness enhancement plan” to secure the competitiveness of the national pharmaceutical industry [29]. The MOHW announced mid- and long-term visions for the realignment of the industrial structure with a focus on several types of innovative pharmaceutical companies: global major pharma, specialized pharma, and global generic pharma. Accordingly, the MOHW designates “innovative pharmaceutical firms” and provides differentiated and customized support, including benefits in the pricing of new medicines, to these firms. For instance, additional drug price rates may be added for a certain period for new drugs that show innovative features. Given this plan, we divided new medicines according to manufacturing type: imported medicines, locally manufactured medicines, and locally developed and manufactured medicines. Then, we assumed that being a locally developed and manufactured medicine was a precondition for shortening the duration.

We sorted new medicines into improved, similar/noninferior, and others, according to the comparative effectiveness, which was determinded by the PBC in the HIRA. We defined improved drugs in terms of clinical effectiveness compared to existing alternatives, and similar drugs were demonstrated to be noninferior or similar to existing alternatives. Others are drugs that are inferior to existing alternatives or for which sufficient information could not be found. Because there would be less controversy in evaluating the clinical- and cost-effectiveness of new medicines that are similar or not inferior to alternatives, we assumed that similar drugs would be promptly adopted by the HIRA. Furthermore, we added the period as an explanatory variable to evaluate a series of policies adopted to reinforce the timely availability of new medicines. Specifically, we separated the period into two parts: the first period, which is before January 2014, and the second period, which is after January 2014.

## Results

### New medicines applied for a reimbursement decision

There have been 128 new medicines approved by the MFDS between 2007 and 2016. We divided these 128 new medicines into the first (2007~2013) and the second period (2014~2016). Appendix presents descriptive statistics of the dependent and explanatory variables used in the model.

Seventy-five out of eight-five (88%) new medicines were designated for reimbursement in the first period. By contrast, forty two out of forty three (98%) new medicines were designated for reimbursement in the second period. We then calculated the duration, which was defined as the time difference between the date of regulatory approval and the date of the reimbursement decision, and subdivided the duration into regulatory approval–reimbursement application and reimbursement application–reimbursement decision. The mean time to reimbursement decision was 29.24 months in the first period, and it ranged from 4.6 months to 128.5 months. While, the mean time to reimbursement decision was 13.40 months in the second period and it ranged from 3.2 months to 42.9 months. However, it should be noted that our observations are right-censored. Next, we divided the duration into regulatory approval–reimbursement trial and reimbursement trial–reimbursement decision. Interestingly, the proportion of each duration out of the total duration had changed. The proportion of the reimbursement trial–reimbursement decision duration out of the total duration was 62.9% (18.39 months out of 29.24 months) in the first period, while, the proportion of the regulatory approval–reimbursement trial duration in the total duration was 64.2% (8.6 months out of 13.40 months) in the second period.

There were eleven and eight new biologics in the first and second periods, respectively, representing an increase from 13% to 19%. The majority of new medicines were imported: 80% and 84% in the first and second periods, respectively. Eleven medicines were locally manufactured and six were locally developed and manufactured in the first period, whereas two medicines were locally manufactured, and five were locally developed and manufactured in the second period. Finally, we sorted the new medicines according to clinical effectiveness. In the first period, 27, 26, and 32 new medicines were sorted as improved, similar, and others, respectively, while, 15, 18, and 10 new medicines were sorted as improved, similar, and others, respectively, in the second period.

### Kaplan-Meier estimates

**Fig. 2:**
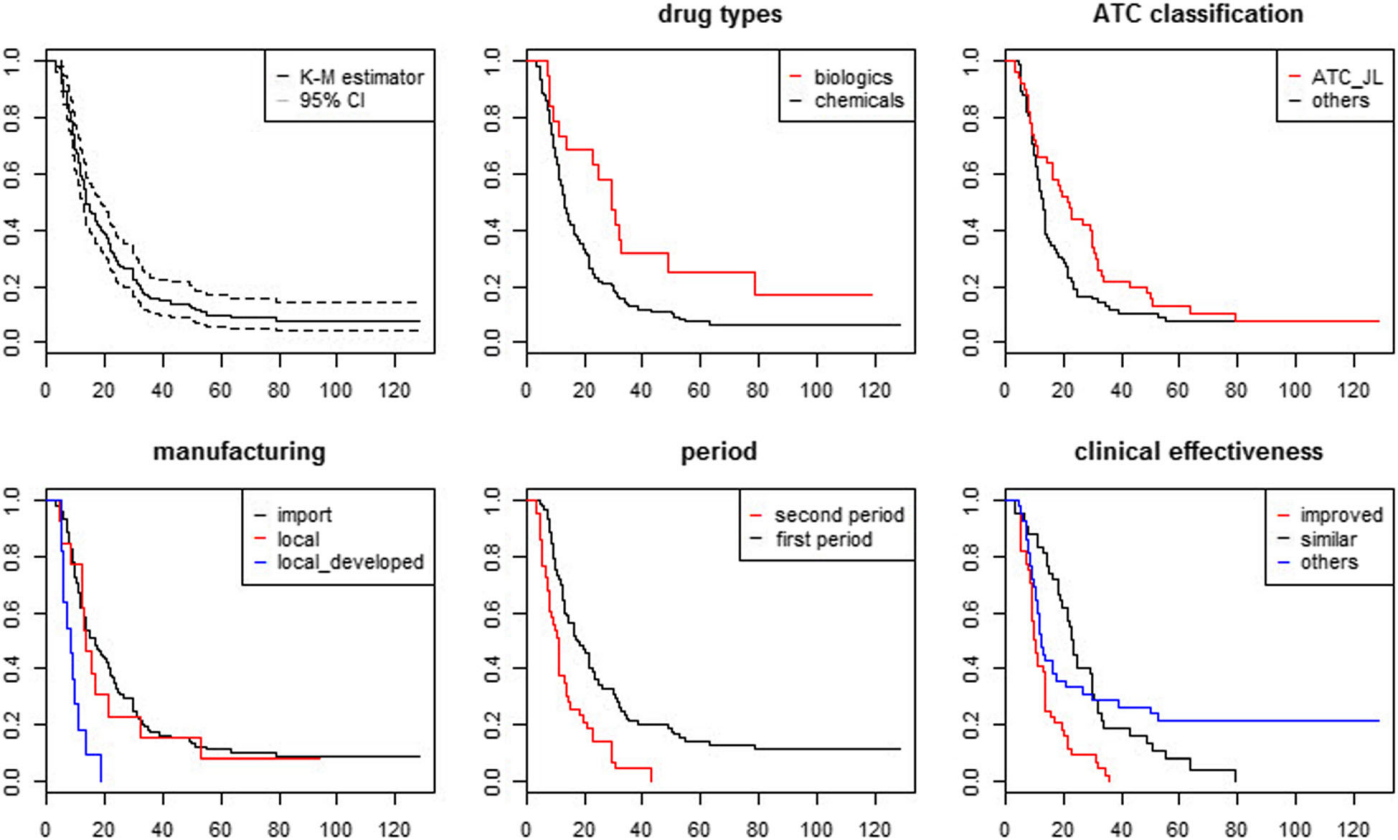
Kaplan-Meier estimates for approval and reimbursement decision

**Fig. 3:**
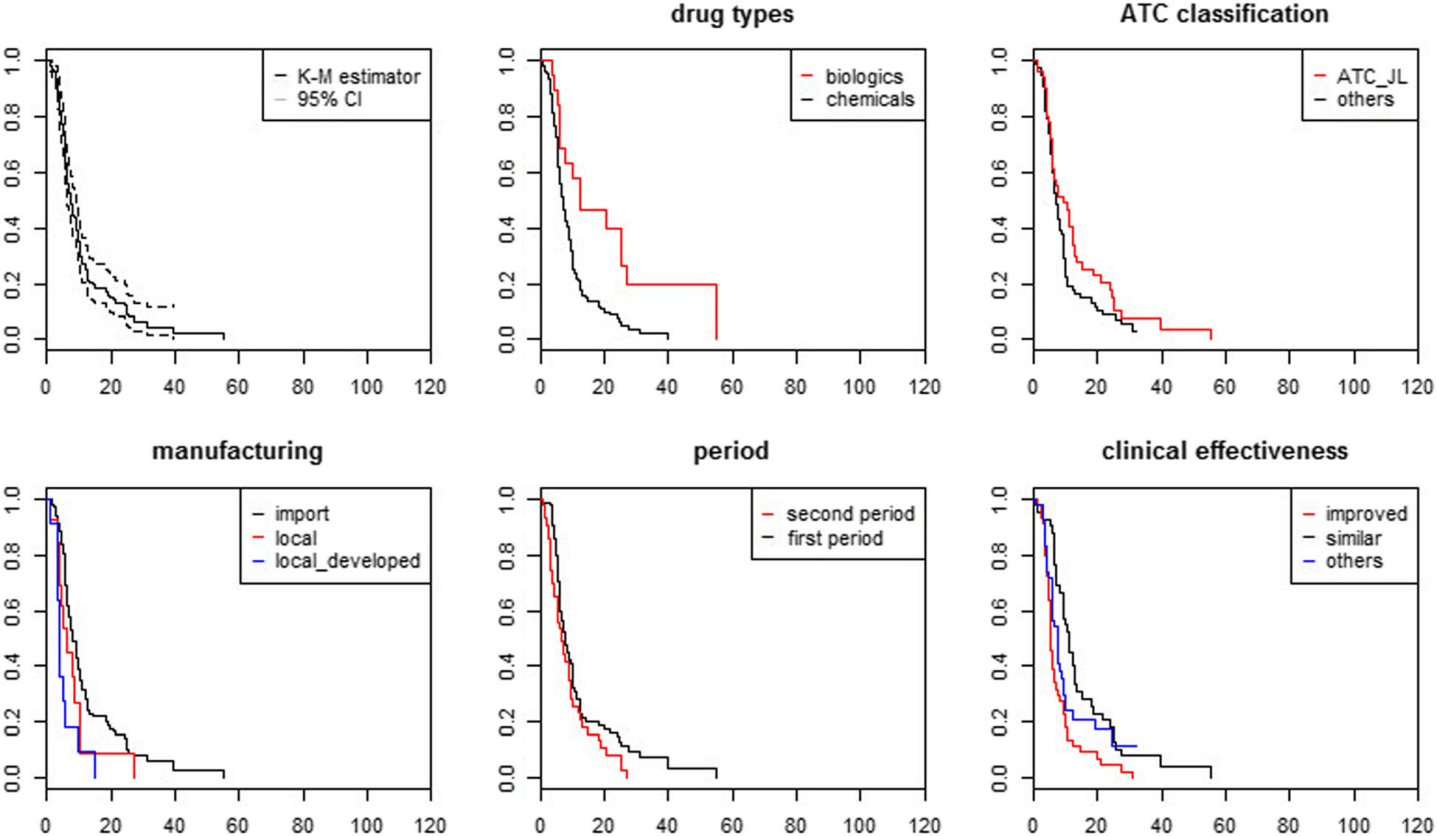
Kaplan-Meier estimates for approval and reimbursement trial

**Fig. 4:**
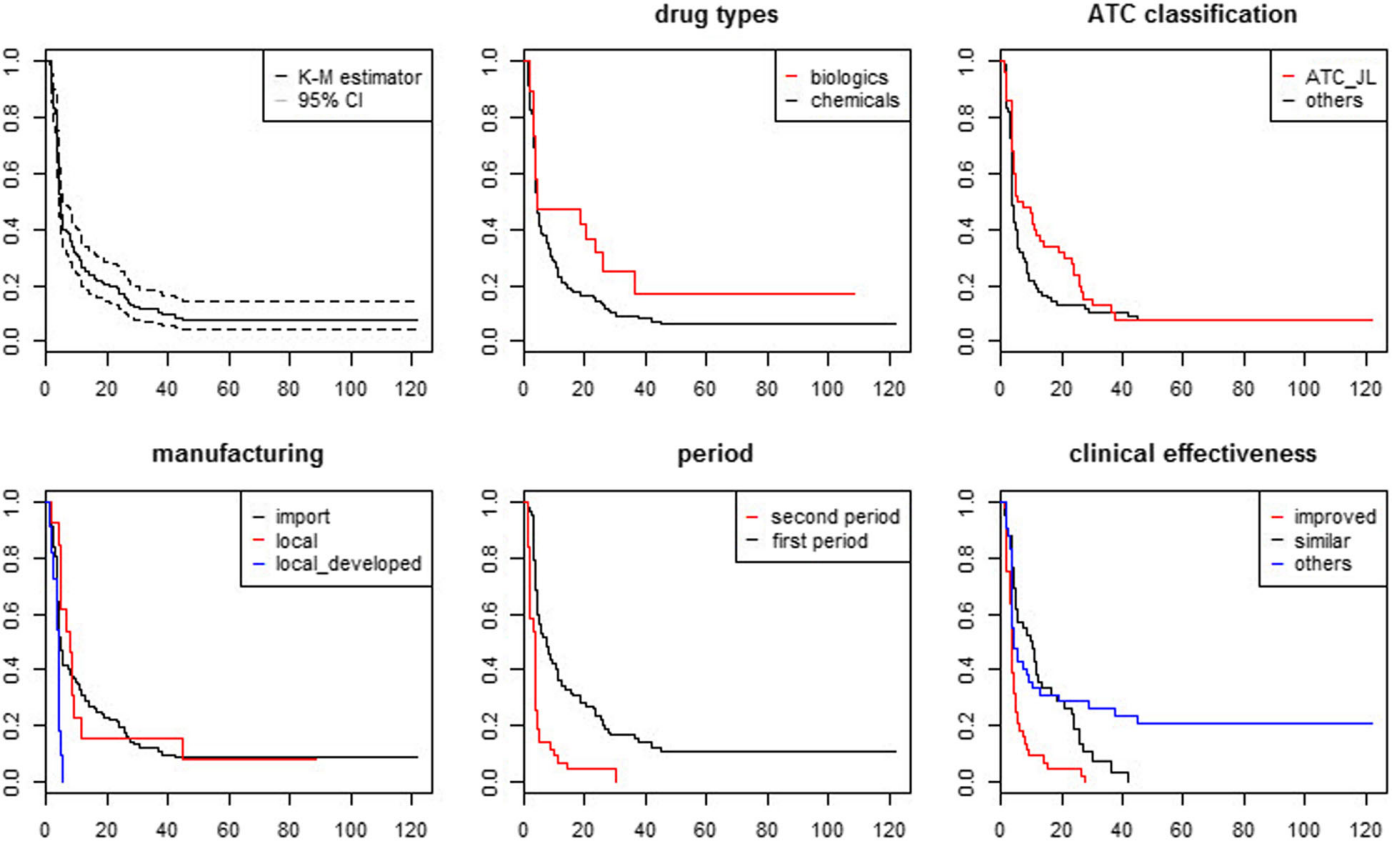
Kaplan-Meier estimates for reimbursement trial and reimbursement decision

Figures 2, 3 and 4 provide a descriptive overview of the differences in the various durations, including regulatory approval–reimbursement decision, regulatory approval–reimbursement application, and reimbursement application–reimbursement decision, using Kaplan-Meier estimates. The estimates indicate the conditional probability that the event will occur after a given period [30]. Specifically, the curve in Fig. 2 indicates the proportion of new medicines that will be reimbursed after a specific period. Therefore, the curve in Fig. 3 indicates the proportion of new medicines for which manufacturers will apply for a reimbursement decision after a specific period.

The first graph in each figure offers a curve without group comparison. The remaining graphs present curves with group comparisons, including drug type, ATC, manufacturing, clinical effectiveness, and the period. We compared the duration by drug types: chemicals and biologics. Figure 2 shows that the duration between regulatory approval and reimbursement decision is short for chemicals. For example, even after 24.53 months from the date of regulatory approval, 57.9% of new biologics remained non-reimbursed, while only 22% of new chemical entities remained non-reimbursed. In addition, the survival difference between the two groups was significant. Second, we compared the duration by the first and second periods. The survival function of the second period group declined rapidly compared to that of the first period group. After 17.30 months from the date of regulatory approval, only half of the new medicines remained non-reimbursed in the first period group. However, 24% of the new medicines remained non-reimbursed at the same time in the second period group. The survival difference between the two groups was also significant.

### Cox proportional hazards model

As the Kaplan-Meier survival estimates are a univariate tool, we also applied a multivariate approach to determine the relative impact of the specific factors on duration: proportional hazards model.

We fitted the Cox model with five discrete factors: manufacturing type, production type, ATC, clinical effectiveness, and the period. Notably, we subdivided the duration into regulatory approval–reimbursement trial and reimbursement trial–reimbursement decision. Table 1 provides interpretations for the specified variables. Note that a negative coefficient indicates a long time to an event while a positive coefficient means a short time to an event. For instance, the time to reimbursement decision for medicines that were developed and manufactured locally was accelerated compared to those of imported medicines (the reference category in the model),. In addition, the second period significantly shortened the duration compared to the first period. However, biologics (reference chemicals), medicines that are improved from the perspective of clinical effectiveness (reference similar medicines), medicines with uncertain clinical effectiveness (reference similar medicines), and those with ATC J or L (reference other classifications) significantly delayed duration in model 1.

**Table 1:** Results for the discrete factor effects and the linear effects from the Cox model with the duration as outcome

Variable	Model 1 (approval_decision)			Model 2 (approval_trial)			Model 3 (trial_decision)			Model 3_1 (trial_decision)		
	Coefficient	Standard Error	*p*-value	Coefficient	Standard Error	*p*-value	Coefficient	Standard Error	*p*-value	Coefficient	Standard Error	*p*-value
Manufacturing types												
locally manufactured (Ref. import)	−0.097	0.317	0.760	0.208	0.326	0.523	−0.276	0.320	0.388	−0.305	0.320	0.340
locally developed and manufactured	1.064	0.349	0.002	0.784	0.338	0.020	0.349	0.355	0.325	0.287	0.356	0.420
Product types												
biologics (Ref. chemicals)	−0.650	0.295	0.027	−0.787	0.310	0.011	−0.316	0.292	0.279	−0.194	0.299	0.515
Clinical effectiveness												
improved (Ref. similar/non-inferior)	−0.597	0.253	0.018	−0.687	0.265	0.009	−0.443	0.259	0.087	−0.338	0.264	0.199
uncertain/others	−0.716	0.252	0.004	−0.320	0.240	0.183	−0.679	0.263	0.009	−0.609	0.265	0.021
ATC												
ATC J or L (Ref. others)	−0.468	0.231	0.042	−0.010	0.242	0.968	−0.520	0.221	0.018	−0.481	0.217	0.026
Period												
the second period (Ref. the first)	1.005	0.223	0.000	0.513	0.212	0.015	1.041	0.221	0.000	0.987	0.221	0.000
Duration												
approval_trial										−0.024	0.015	0.099

## Discussion

Timely regulatory approval and pricing and reimbursement decisions are necessary for new drugs to be accessible. We aim to analyze the duration from regulatory approval to reimbursement decision for new drugs in the Korean market and to evaluate various factors that affect the timely availability of new medicines. Furthermore, we subdivided the duration into regulatory approval–reimbursement application and reimbursement application–reimbursement decision to understand the adoption of new drugs decided by several stakeholders in the market, including the strategic behavior of manufacturers.

### Summary of findings

There are some interesting findings that should be noted in our duration model. First, a series of policies that were introduced to reinforce access to medicines after 2014 was effective in improving the timely availability of new medicines. Specifically, the second period shortened all durations in the models, including approval–decision, approval–application, and application–decision. This result indicates that policies led manufacturers to apply for reimbursement earlier, and the authorities, including the HIRA, the NHIS, and the MOHW, to more promptly offer a favorable decision.

Second, biologics (reference chemicals), improved medicines and medicines that are uncertain from the perspective of clinical effectiveness (reference similar medicines), and medicines belonging to ATC J or L (reference other classifications) presented significant delays in the duration between regulatory approval and reimbursement decision (or in model 1). However, different patterns were presented in models 2 and 3. For instance, biologics and improved medicines experienced delays in the duration between regulatory approval and reimbursement trial. This result indicates that these factors influenced the manufacturer’s strategic decision on applying for the reimbursement trial. In other words, manufacturer may unintentionally or intentionally delay the application due to either preparing the dossiers submitted to the HIRA or strategically considering that Korea is a low-price market and external referencing price in other markets. However, uncertain drugs from the perspective of clinical effectiveness and ATC J or L delayed the duration between reimbursement trial and reimbursement decision. These factors require the HIRA to prolong the time taken to evaluate the submitted dossiers and to make a favorable decision. Sometimes, the NHIS might need more time to negotiate the final price of these new medicines.

Third, medicines that were developed and manufactured in the local market were adopted promptly. Specifically, this factor significantly decreased the duration between regulatory approval and the reimbursement trial. However, the duration between the reimbursement trial and the decision was not significantly shortened by this factor.

### Comparison with other studies

Bae et al. (2016) evaluated the reimbursement recommendation of the PBC for medicines that applied for reimbursement decisions between 2007 and 2014 [10]. There had been 253 drugs submitted to the HIRA for a reimbursement decision. It should be noted that Bae et al. (2016) included only final submissions and recommendations in the study when manufacturers submitted the dossiers repeatedly, in the case of rejection by the HIRA or the failure of price negotiations after a positive recommendation by the HIRA. Of these 253 drugs, 175 (69.2%) were recommended for listing, and 78 (30.8%) were rejected. Our study interest is in the duration from regulatory approval to reimbursement decision of drugs that were designated as new medicines by the MFDS between 2007 and 2016, which is the reason there is a smaller number of subjects in our study compared to the number in the study by Bae et al. (2016). Although there were some differences in the subjects of analysis, we can conclude that the acceptance rate has increased.

Additionally, Bae et al. (2016) presented the decision of HIRA considering clinical effectiveness. The acceptance decision rate for clinically noninferior/similar drugs (74.1%) was higher than that of clinically improved drugs (67.6%) [10]. The authors suggested that this observation could be explained by the lower price submitted for clinically noninferior/similar drugs compared to that for existing drugs. In addition, if the evidence on the clinical effectiveness of drugs is uncertain, it is less likely to be accepted (15.4%). These findings are consistent with our observations: noninferior/similar drugs have the shortest duration between regulatory approval and reimbursement decisions. Given these results, we could conclude that drugs considered uncertain in the perspective of clinical-effectiveness require the related authorities, including the HIRA, the NHIS, and the MOHW, to take more time to make a favorable decision.

### Study limitations

This study has several limitations. First, there is a possible limitation in our methodology. Because of the unavailability of information on the reimbursement application date, we used the date of the PBC appraisal as a proxy for the reimbursement application date. Second, this study noted the first trial for reimbursement application. Therefore, if a manufacturer produces additional data on clinical effectiveness, the clinical effectiveness of the drug may change over time. In addition, there were several cases in which the PBC reviews on the clinical effectiveness of the drug were ambiguous or incomplete. To address these problems, the author and other person independently evaluate the information in the PBC reviews and reached a consensus on the clinical effectiveness.

## Conclusions

In this study, we calculated the duration from regulatory approval to reimbursement decision of new medicines in the Korean market, subdivided the duration into regulatory approval–reimbursement application and reimbursement application–reimbursement decision, and applied an event history model to evaluate various factors affecting the timely availability of new medicines. Between 2007 and 2016, a total of 128 new medicines were designated by the MFDS and applications submitted for reimbursement decisions. Delays in access to new medicines occurred at various levels, and various factors were affected in the different durations. Given a series of policies introduced to reinforce access through the timely availability of new medicines, we separated the period into two parts. The duration between regulatory approval and reimbursement decision has decreased, and the main cause of the delay has changed. For instance, the proportion of reimbursement trial–reimbursement decision in the total duration was 62.9% (18.39 months out of 29.24 months) in the first period, while the proportion of regulatory approval–reimbursement trial in the total duration was 64.2% (8.6 months out of 13.40 months) in the second period. These policies to reinforce access to medicines after 2014 has been effective in improving the timely availability of new medicines, including both manufacturers promptly submitting their reimbursement application and a timely review process by the authorities.

## Appendix

**Table 2:** Characteristic of new medicines applied for reimbursement decision between 2007 and 2016

	2007–2013 First period (*n* = 85)	2014–2016 Second period (*n* = 43)
Reimbursement		
yes	75	42
no	10	1
Duration ^a, b^ (right censored)		
approval_reimbursement decision	29.24, (28.56)	13.40, (9.66)
approval_reimbursement trial	10.85, (9.32)	8.60, (6.77)
reimbursement trial_ reimbursement decision	18.39, (25.33)	4.79, (5.73)
Duration (reimbursed drugs only)		
approval_reimbursement decision	20.61, (14.82)	12.83, (9.04)
approval_reimbursement trial	9.95, (8.77)	8.50, (6.82)
reimbursement trial_ reimbursement decision	10.66, (10.34)	4.33, (4.92)
Product types		
chemicals	74	35
biologics	11	8
Manufacturing types		
import	68	36
local manufacturing	11	2
local development and manufacturing	6	5
ATC		
ATC J or L	29	21
others	56	22
Clinical effectiveness		
improved	27	15
similar/non-inferior	26	18
others	32	10

^a^ unit: months

^b^ mean (standard deviation)

## Abbreviations

ATC: the Anatomical Therapeutic Chemical Classification
HIRA: the Health Insurance Review and Assessment Service
ICER: Incremental cost-effectiveness ratio
Korea: the South Korean government
MFDS: the Ministry of Food and Drug Safety
MOHW: the Ministry of Health and Welfare
NHIS: the National Health Insurance Service
PBC: the Pharmaceutical Benefits Committee
Plan: Drug expenditure rationalization plan
PLS: A positive list system
